# Removal of MTBE and BTEX Pollutants from Contaminated Water Using Colloidal Activated Carbon (CAC)

**DOI:** 10.1021/acsomega.4c06974

**Published:** 2024-12-26

**Authors:** Abdullah Basaleh, Amjed Hassan, Bassam Tawabini, Mohamed Mahmoud, Fahad Alghamdi, Abdulmogni Althubiti, Muhammad Alrayaan, Rayan Al-Nasser

**Affiliations:** † Geosciences Department, College of Petroleum Engineering and Geosciences, King Fahd University of Petroleum & Minerals (KFUPM), P.O. Box 5070, Dhahran 31261, Saudi Arabia; ‡ Center for Integrative Petroleum Research, College of Petroleum Engineering and Geosciences, King Fahd University of Petroleum & Minerals (KFUPM) institution, P.O. Box 5070, Dhahran 31261, Saudi Arabia; § Petroleum Engineering Department, College of Petroleum Engineering and Geosciences, King Fahd University of Petroleum & Minerals (KFUPM) institution, P.O. Box 5070, Dhahran 31261, Saudi Arabia; ∥ Groundwater Protection Unit, Environmental Department, Saudi Aramco, P.O. Box1977, Dhahran 31311, Saudi Arabia

## Abstract

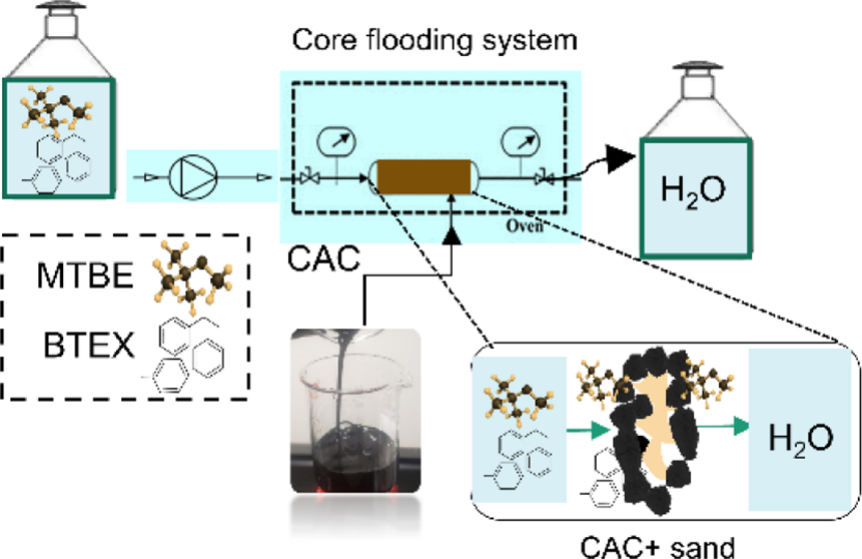

Methyl tertiary-butyl ether (MTBE) and BTEX (benzene, toluene, ethylbenzene, and xylenes) are common groundwater contaminants that pose significant health risks. This study investigated the efficiency of a colloidal activated carbon (CAC) material in removing MTBE and BTEX from contaminated water using batch and continuous core flooding systems. In the batch system, a mixture of sand and carbonate was coated with 1–3 g of CAC for the removal of contaminants. The core flooding system was packed with similar materials and mixed with 10 g of CAC. X-ray diffraction (XRD) revealed that the carbonate consists of more than 97% calcite, with traces of clay and quartz minerals. On the other hand, the sandstone sample showed around 89% quartz, 8% clay minerals, and traces of feldspar. The CAC has a surface area of 1050 m^2^/g, mean particle size of 31.8 μm, density of 1.0976 (g/cm^3^), viscosity of 24.4 mPa·s, and a negative surface charge. Spiked water samples with 2000 ppb MTBE and BTEX concentrations ranging between 1000 and 200 ppb were injected through the system at rates of 0.5 and 1.0 mL/min. The results showed that the type of packing materials and flow rate have a significant impact on contaminant removal. For example, the removal efficiency was higher in sandstone due to uniform particle shapes facilitating better water distribution and CAC accessibility within the pore medium. A lower injection rate (0.5 mL/min) resulted in higher removal efficiencies due to increased contact time between contaminants and CAC. At 1 mL/min, the maximum removal of 94% and 65% for MTBE and benzene was achieved in carbonate, while 99% and 96% were achieved in sandstone, respectively. At 0.5 mL/min, complete MTBE removal and over 95% benzene removal were achieved in both materials. Overall, the CAC demonstrated excellent MTBE and BTEX removal capabilities, exceeding 95%, offering a promising approach for in situ groundwater remediation.

## 1 Introduction

MTBE and BTEX are synthetic organic compounds that have found widespread use in various industries and commercial applications. This has led to their dissipation as environmental contaminants. MTBE is primarily used as an oxygenate in gasoline to reduce carbon monoxide emissions from vehicles and increase the octane rating.^1,2^ Similarly, BTEX compounds are a class of volatile organic compounds found in gasoline and other petroleum-derived products; they are used as solvents, in the production of paints, plastics, and numerous industrial processes.^3,4^

The main sources of MTBE and BTEX dissipation in the environment include gasoline spills, leaks from underground storage tanks, transfer pipes, and improper disposal methods.^5,6^ Precipitation infiltration and dispersion from urban areas can also act as nonpoint sources. MTBE and BTEX contamination have been reported in shallow groundwater near major metropolitan regions in the United States, such as Colorado, Los Angeles, New York, and Texas.^7−12^ Chemical plants, refineries, and other manufacturing facilities have also been identified as hotspots for MTBE and BTEX contamination. For instance, in 1994, a 42 000 L gasoline spill in Ronan, Arizona, resulted in the contamination of the local groundwater aquifer with MTBE and BTEX, persisting in the shallow groundwater eight years after the incident.^13^ Moreover, in 1998, Fogg et al.^14^ reported that there are 32 779 sites contaminated with chemicals from underground storage tanks in California, where 90% of these sites are contaminated with hydrocarbons. Despite being banned in some countries, MTBE remains the second most frequently reported volatile organic compound in shallow groundwater surveys in the United States.^15,16^ The concentration of MTBE was above the drinking water advisory limit by the USEPA (20 μg/L) in 3% of the samples. Similarly, studies have reported BTEX concentrations in groundwater exceeding drinking water MCLs in up to 90% of samples from oil spill sites.^12^ Moreover, MTBE has also been reported in a recent survey of 50 gasoline-impacted sites in Europe.^11^ In addition, a recent study conducted by Camaj et al.^17^ reported the presence of BTEX in the White Drin River, Kosovo, which was ascribed to the high traffic density near the river.^18^

The dissipation of MTBE and BTEX in the environment can have significant adverse effects on human health and the ecosystem. MTBE is suspected to be carcinogenic and genotoxic, and its presence in an aquatic environment may cause unpleasant odor and skin and eye irritation.^18^ BTEX is known to be toxic, with the potential to cause neurological, developmental, and reproductive effects. Exposure to toluene may cause kidney and liver damage. It may also cause cardiovascular effects and neurological system symptoms. Neurological and developmental system effects are also reported with exposure to ethylbenzene. Xylene may cause renal symptoms and neurological, hepatic, and developmental symptoms. Benzene has been classified as a carcinogenic compound by the IARC (IARC, 2020).^4^ Benzene may also cause acute myeloid leukemia and aplastic anemia. Prolonged exposure to benzene may cause several neurological symptoms, including headaches, drowsiness, confusion, and tremors, as well as eye and skin irritation.^19^ Ethylbenzene is considered a suspected carcinogen.^20^

Physical, chemical, and biological methods have been employed in the remediation of MTBE and BTEX.^15,21^ MTBE and BTEX removal were investigated using various methods, including advanced oxidation, air stripping, membrane separation, and aerobic biodegradation.^22,23^ Despite these efforts, MTBE presents challenges due to its low Henry’s constant, high solubility in water, and small molecular size, rendering it highly mobile in the environment and relatively resistant to biological degradation, thus limiting the effectiveness of biological methods. Moreover, MTBE removal by air stripping is difficult due to its low volatility and high solubility. Furthermore, chemical oxidation methods may generate undesirable byproducts such as bromate and tertiary-butyl alcohol.^24^ Adsorption is a process that can be used in situ (through permeable reactive barriers) or ex-situ and can produce quite excellent removal efficiencies. The ex-situ adsorption process has been widely used for MTBE and BTEX removal using numerous adsorbents such as diatomite,^25,26^ zeolitic materials,^5^ graphene-iron nanocomposites,^27^ and carbon nanotubes.^28^ Because of its high adsorption capacity, carbon is a widely utilized adsorbent for organic molecules, including MTBE and BTEX.^22,28−37^ However, these studies used granular or powdered carbon for ex-situ treatment, which is costly for groundwater remediation. Consequently, there is a growing focus on in situ groundwater treatment. For in situ adsorption, the adsorbent is deployed in the subsurface through injection to establish permeable reactive barriers, necessitating the activated carbon to be in a colloidal stable suspension. Several studies have reported on the use of CAC for in situ remediation of groundwater contaminated with various contaminants, including chlorinated solvents,^38−40^ and PFAS.^40−43^ In light of the above, previous studies on the remediation of groundwater contaminated with MTBE and BTEX using CAC have been very limited. To the best of our knowledge, only one study investigated CAC for MTBE and BTEX removal. Alshahrani et al.^44^ investigated CAC and iron-modified Fe-CAC for MTBE and BTEX removal in batch suspension experiments. The CAC was mixed with various substrates, including limestone, sand, and a 1:1 mixture of limestone/sand. They also used granular activated carbon (GAC) for comparison. It was found that mixing CAC with different substrates did not improve the removal efficiency of MTBE, while a slight improvement was noticed for benzene and toluene removal. The maximum removal efficiencies by CAC and Fe-CAC were 40% and 20% for BTEX and MTBE, respectively. Furthermore, the researchers noted that variation in the salinity and pH of the treated water had an insignificant impact on the MTBE and BTEX removal efficiencies.

The petroleum industry usually uses laboratory core flooding experiments to assess the extent of oil that can be recovered from a reservoir. In these experiments, a core pack, often unconsolidated sand particles^45−47^ or, in some cases, consolidated cores such as sandstone and carbonate outcrops,^48−51^ mimicking the porous natural reservoir, is flooded with an injection fluid, and the resultant output in terms of oil recovery is measured. Several core flooding systems have been used for different applications, including enhanced oil recovery,^52^ carbon dioxide sequestration (CO_2_),^53^ monitoring fluid migrations,^54^ and gas saturation monitoring.^55^

To gain a better understanding and estimation of the CAC-injected space distribution and migration processes, as well as the relative permeability of multiphase flow in porous media and the phase status of the MTBE-BTEX plume, laboratory core flooding experiments, similar to those used in the petroleum industry, could be more effective. Core flooding is widely used to determine the permeability and interaction between soil formation and various liquids. Therefore, it was utilized to evaluate CAC for MTBE and BTEX removal from contaminated groundwater.

In the current study, a core flooding technique was utilized to simulate the groundwater flow through injected CAC to assess the efficiency of the CAC for MTBE and BTEX removal from contaminated water. This study is essential for the large-scale injection of CAC. The core flooding system is suitable for (1) CAC injection in different subsurface geological formations, namely, carbonate and sandstone, (2) assessing the efficiency of the CAC for MTBE and BTEX removal from contaminated subsurface water in different geological formations, and (3) investigating the removal behavior of MTBE and BTEX from the subsurface by CAC at different flow rates.

In light of the above, the use of CAC in most previous studies requires the injection of electron donors to stimulate the biological degradation of contaminants in addition to the removal by an adsorption mechanism, which is costly and time-consuming. However, in this study, we only investigated adsorption as the primary process for groundwater remediation, which will save time and cost. To the best of our knowledge, the use of core flooding systems to simulate in situ groundwater treatment has not been reported. Therefore, the current investigation will open a new research trend. The performance of a new CAC in removing MTBE and BTEX from water is examined for the first time in a continuous flow core flood system. Different types of formations were utilized to evaluate the influence of the rock mineralogy on the contaminant removal efficiency. Also, the impact of the water flow rate on the removal of MTBE and BTEX from water was determined by using different injection rates. Gas chromatography (GC) analysis was performed on the injected and produced fluids to measure the concentrations of MTBE and BTEX, and then the removal efficiencies were calculated.

## 2 Materials and Methods

### 2.1 Materials

Chemicals used in this study include MTBE (p.a.; ≥99.5% GC), benzene (≥99.5% GC), toluene (ACS, ≥99.5), a xylene mixture (80% xylene and 20% ethylbenzene), and methanol (HPLC, ≥99.5%). The xylene fraction consists of equal concentrations of *p*-, *m*-, and *o*-xylene. The DI water was obtained using a water purification system. A stock solution mixture was prepared by dissolving MTBE and BTEX in methanol at a concentration of 1000 mg/L, with individual component concentrations of 1000 mg/L for MTBE, 500 mg/L for benzene and toluene, 300 mg/L for the xylene mixture, and 75 mg/L for ethylbenzene. For each experimental run, a spiked solution mixture was freshly prepared by diluting the stock mixture in DI water to obtain the following concentrations: 2000 μg/L for MTBE, 1000 μg/L for benzene and toluene, 600 μg/L for the xylene mixture, and 150 μg/L for ethylbenzene. The diluted mixture was stirred for 3 h to ensure complete dissolution of all compounds. The pH of the mixture was maintained at a value of around 6 throughout the experiment.

The characteristics of the CAC used in this study are depicted in Table 1. Carbonate and sandstone formations were used as representative geological formations. The mineralogical composition of the samples used was determined using XRD analysis. The carbonate formation showed around 97% calcite and some traces of clay and quartz minerals. The sandstone samples showed 89% quartz, 8% clay, and traces of feldspar.

**Table 1:** Characteristics of the CAC Used in This Study

Character	Value
solution density	1.0976 (g/cm^3^)
median diameter of the adsorbing material (Wet)	31.5 μm
viscosity (room temperature 25 °C)	≈24.4 mPa.s.
surface area (BET) from the manufacturer	1050 m^2^/g

### 2.2 Analytical Method

MTBE and BTEX concentrations were measured using GC/MS following EPA Method 8260^56^ as reported by Alshahrani et al.^44^ The headspace analyzer was coupled to the GC/MS for automated analysis. 1 mL of the solution was collected in 5 mL vials at each sampling interval and transferred to the GC/MS for analysis. The GC/MS had a headspace injection unit, which was used to incubate solutions in an agitator for 5 min at 90 °C before injecting volatile components directly into the GC column using a headspace syringe heated to 120 °C.

### 2.3 Characterization of CAC

CAC was dried in an oven at 110 °C for 2 h, crushed, and kept in an airtight container for various characterizations. The surface morphology and elemental compositions were visualized by using SEM coupled with EDXS (JEOL JCM-7000 BENCHTOP). The zeta potential at different pH values (2–12) was measured using an Anton Paar Litesizer 500 at 25 °C. A background electrolyte of KNO_3_ at a concentration of 10 mM was employed. To prepare solutions for zeta potential measurement, 30 mL of KNO_3_ was placed in 50 mL conical flasks. Subsequently, the pH levels were adjusted to values of 2, 4, 6, 8, 10, and 12 using diluted solutions of NaOH and HNO_3_. Following the pH adjustments, 0.1 g of CAC was added to each flask. To ensure the equilibrium interactions between the CAC and the solution, the mixture was shaken and left overnight. Subsequently, the samples underwent centrifugation, separating any precipitates, and the zeta potential was measured. The FTIR spectrum was recorded using the KBr pellet method with an FTIR spectrometer from Bruker. The density and dynamic viscosity of the CAC aqueous solution were measured by using a liquid densitometer. Also, the particle size distribution was determined by using a laser-diffraction-based wet particle size analyzer.

### 2.4 Batch Experimental Work

A series of batch experiments were conducted to investigate the efficiency of CAC for the adsorption of MTBE and BTEX from water. Various dosages of CAC (1, 2, and 3 g) were mixed with 20 g of a soil mixture composed of sand and carbonate (1:1 w/w ratio). These mixtures were placed in 45 mL clear open-top volatile organic analysis (VOA) vials, which were then filled to the brim with a solution of MTBE and BTEX, ensuring no headspace to minimize volatilization. The vials were subsequently placed on a horizontal shaker at a shaking speed of 150 rpm. Samples were collected at different time intervals using syringes, filtered, and immediately capped tightly for GC analysis to determine the concentrations of MTBE and BTEX. Additionally, a control experiment (with soil only) was conducted under similar conditions to evaluate the effect of adsorption by the substrate soil and volatilization during the experiments.

### 2.5 Core-Flooding Experimental Work

**Figure 1:**
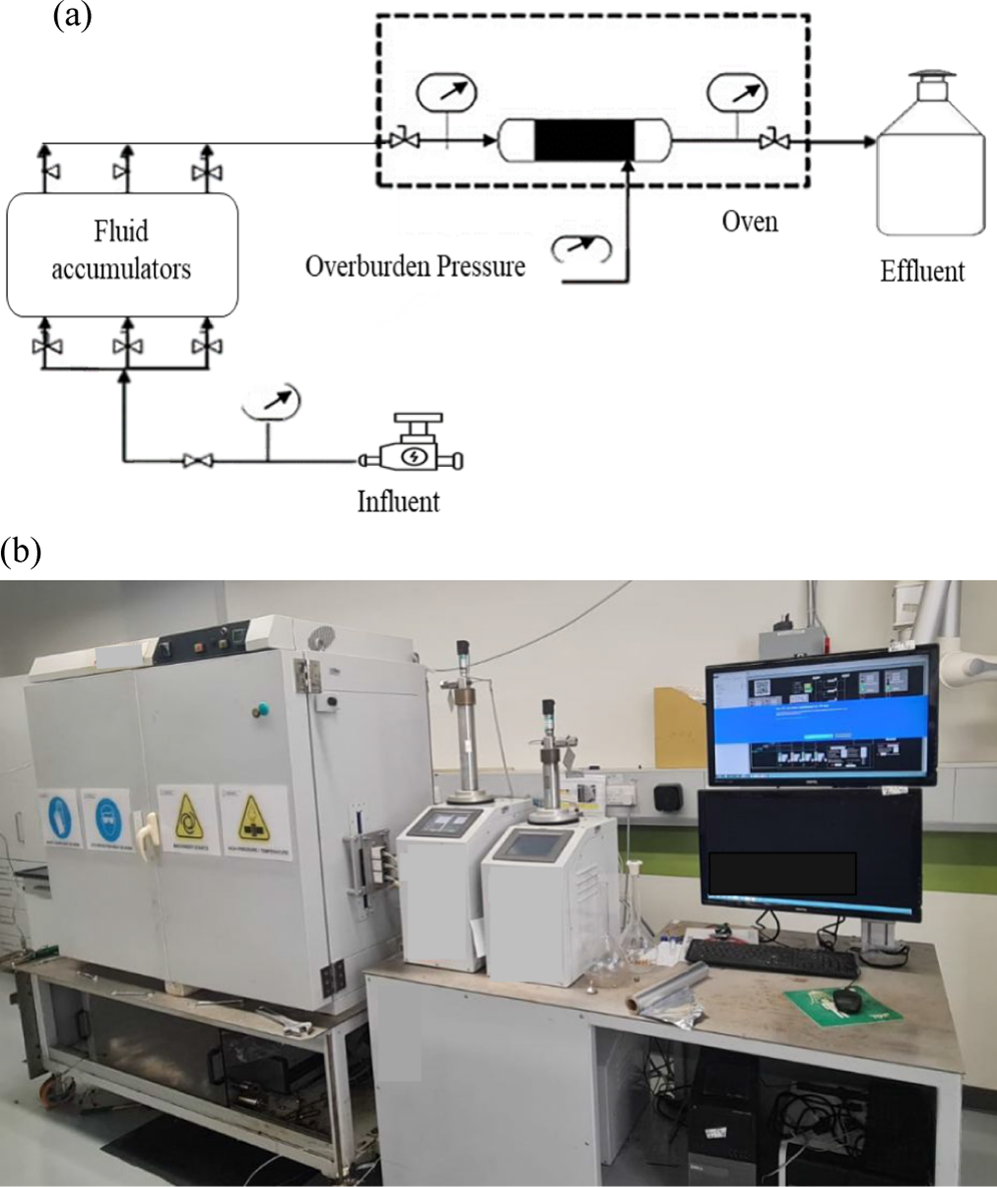
Core flooding setup used in the current study: (a) schematic diagram and (b) the real setup.

Eight laboratory-scale flooding experiments were carried out using carbonate and sandstone samples. A state-of-the-art core flooding setup was employed, encompassing an injection pump, transfer cells, pressure groups, backpressure, and a core holder. The core measured 10 cm in length and 2.5 cm in diameter. Schematic and real representations of the core flooding setup are presented in Figure 1, illustrating the key components utilized in this investigation. Two primary types of flooding experiments were conducted.

The colloidal activated carbon (CAC) was injected first to deposit inside the core and act as a filter. A backpressure of up to 400 psi was applied, and a very small flow rate of around 0.1 cm^3^/min was used to ensure a good and uniform distribution of the injected fluids along the pore system. All measurements were carried out at room temperature. After injecting the CAC into the packed core, the prepared water solutions were injected at different flow rates; injection rates of 0.5 and 1 cm^3^/min were used. The seepage velocity for each injection rate was calculated based on the porosity of the geological formation. The porosity of sandstone used in this study is 0.26, while that of carbonate is 0.22. In sandstone, the seepage velocities were 0.4 (5.76 m/day) and 0.8 cm/min (11.52 m/day) at injection rates of 0.50 and 1 cm^3^/min, respectively. Similarly, in carbonate, the seepage velocities were 0.46 cm/min (6.62 m/day) and 0.92 cm/min (13.29 m/day) at injection rates of 0.50 and 1 cm^3^/min, respectively. For comparison, groundwater velocities in sand aquifers typically range from 0.1 to 1 m/day, while they can reach up to 10 m/day in sand and gravel aquifers.^57^ The flow rates used in this study are close to the typical groundwater velocities observed in sand and gravel aquifers.

The flow rates were controlled using an injection pump, and all fluids were injected from high-pressure accumulators to ensure no vaporization of the volatile components. Furthermore, control experiments were performed where no CAC was used. These control experiments were used as a benchmark to describe the losses in hydrocarbons within the system. All control experiments showed very acceptable losses, where the hydrocarbon content was very close to the injected solution. Thereafter, the experiments were carried out by injecting CAC and then water solutions. In addition, the produced effluent was collected by using sealed tubes to avoid volatilization. Around 30 mL of the injected fluids were collected over a period of 30 min to 1 h, depending on the applied flow rates.

## 3 Results and Discussion

### 3.1 CAC Characterization

**Figure 2:**
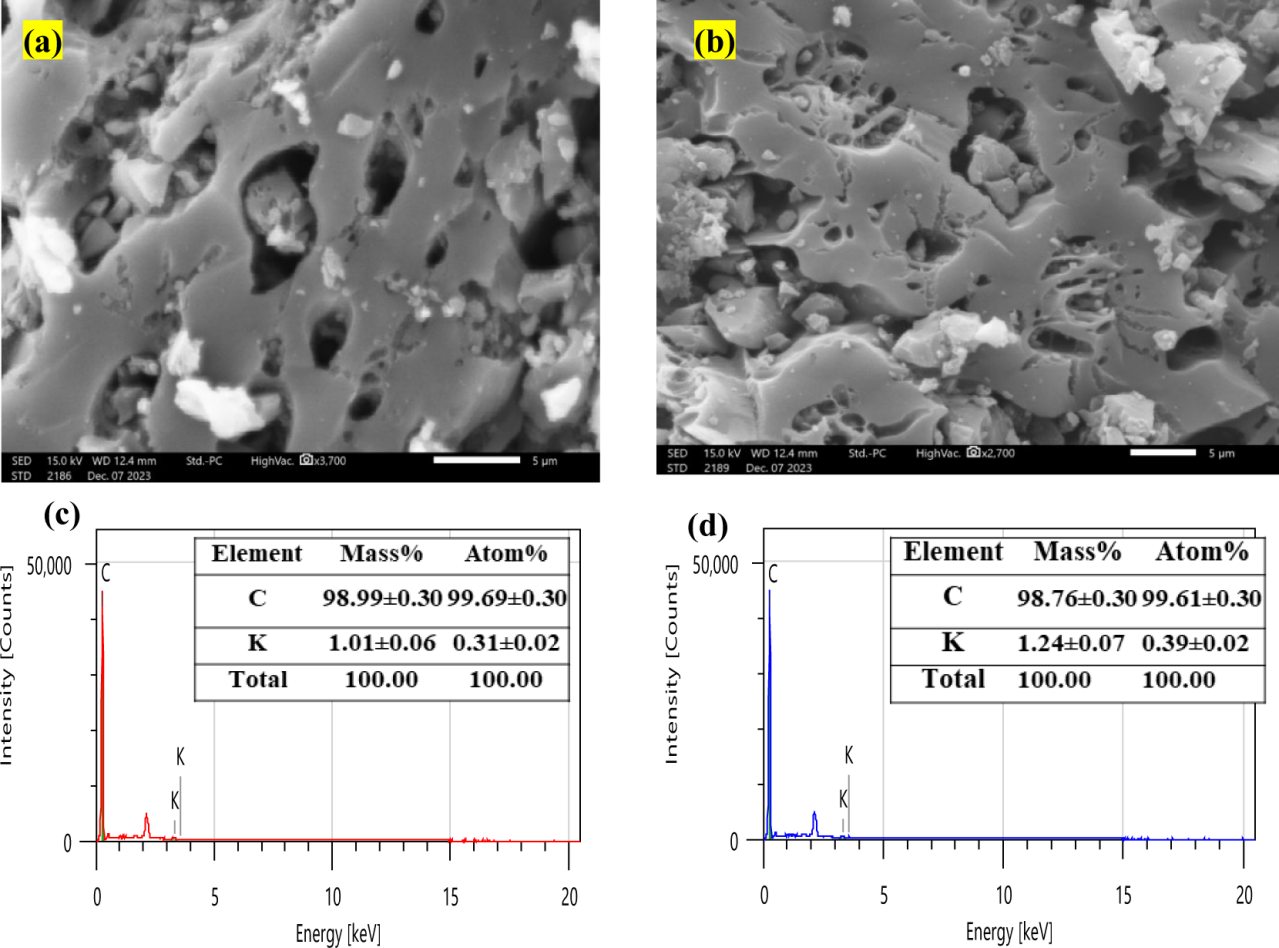
Surface morphology and elemental compositions of the CAC: (a,b) SEM microphotographs and (c,d) chemical compositions derived from the EDXS spectra.

**Figure 3:**
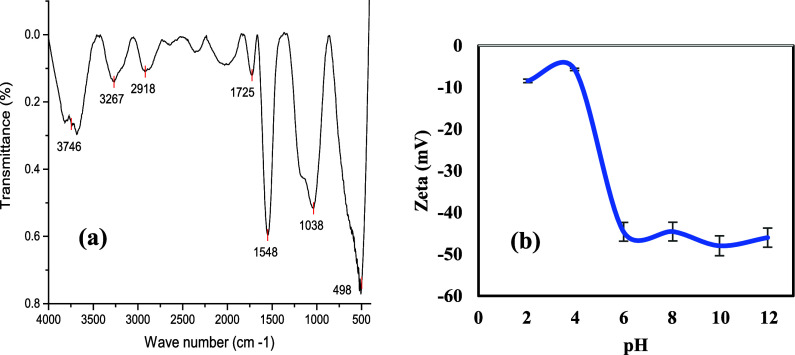
CAC characterizations: (a) FTIR spectrum and (b) zeta potential measurements.

Figure 2 depicts the SEM/EDX analysis of the CAC. The presence of many pores, cracks, and cavities with varied shapes and sizes is revealed by SEM images, indicating the porous nature of CAC. According to the EDX analysis, carbon is the predominant component, accounting for around 99%, with a trace quantity of potassium accounting for 1%. These findings are consistent with previous studies of activated carbon materials, emphasizing their porous structures and elemental compositions. The FTIR spectrum of the CAC shows several characteristic bands, as depicted in Figure 3a. The broad peak at 3746 cm^–1^ can be attributed to the vibrations of the hydroxyl (−OH) groups. The broad peaks observed at 3267 and 2918 cm^–1^ might be ascribed to the stretching of carboxyl (−COOH) groups. Furthermore, the peak at 1725 cm^–1^ can be assigned to the C=O stretching vibration of the carboxyl group. The prominent peak at 1548 cm^–1^ is attributed to the C=C stretching vibrations, signifying the presence of unsaturation in the molecular structure. The prominent peak at 1038 cm^–1^ can be ascribed to the C–O stretching, which is consistent with the presence of oxygen functional groups. The peak at around 498 cm^–1^ is ascribed to the bending vibrations of the carbon–hydrogen (C–H) bonds.

The zeta potential of the CAC is depicted in Figure 3b. Notably, all pH levels have consistently negative zeta potential values, indicating that the CAC surface is negatively charged, which can be attributed to the presence of oxygen functional groups, such as carboxyl and hydroxyl, on the surface. Similar findings have been reported in the literature, where the zeta potential of the material was found to be negative across the entire pH range.^60−63^ This is advantageous for the CAC, as the negative charge will maintain the material in a stable colloidal suspension regardless of the solution pH. The repulsions between the negatively charged CAC colloids prevent their agglomeration and settling, which is desirable for CAC subsurface applications, where a stable colloidal suspension is required. Moreover, the findings show that the negative surface charge of the CAC increases as the pH of the solution increases. This could be ascribed to the ionization of the functional groups on the CAC surface. Functional groups such as carboxyl (−COOH) may deprotonate to generate carboxylate (−COO−) ions at higher pH values, leading to an increased negative surface charge. The increased negative surface charge of CAC at higher pH levels is relevant since it implies that CAC, regardless of pH, has a greater potential for adsorbing cationic ions. Positively charged ions, such as cationic species, can be attracted to the negatively charged surface of the CAC through electrostatic interactions. Moreover, since the CAC is injected in the saturated zone where the pH of groundwater is near neutral, the negative charge of the CAC at these pH values enhances interactions with aromatic hydrocarbons such as BTEX. The oxygen atoms of the carboxyl on the CAC surface act as electron donors, while the aromatic rings of BTEX act as electron acceptors,^64^ resulting in higher adsorption. Moreover, MTBE is a weakly positive compound, which enhances electrostatic interactions between the CAC negative charge and MTBE.

In addition, the negative surface charge of the CAC will allow better distribution of the CAC in the subsurface due to the electrostatic repulsion between the CAC and the predominant surface of silica in the subsurface.^65^ The pH of the solution in all experiments was unadjusted and remained consistently at a value of around 6, where the negative zeta potential value is very high at this pH, allowing high π-interactions between the adsorbent and adsorbate. The particle size distribution of the CAC is shown in Table 2. The findings show that the median particle size, represented as *d*_50_, is 31.85 μm. This means that 50% of the CAC particles have a diameter of 31.85 μm or less. Furthermore, the data show that the *d*_90_ value is 74 μm, indicating that 90% of the CAC particles have a size less than or equal to 74 μm. Furthermore, the *d*_10_ value is 9.65 μm, indicating that 10% of the CAC particles have a diameter of 9.65 μm or less.

**Table 2:** Particle Size Distributions of the CAC

Percentile	Particle Size (μm)
*d*_10_	9.65
*d*_50_	31.85
*d*_84_	62.33
*d*_90_	73.99

### 3.2 Batch Adsorption Study

**Figure 4:**
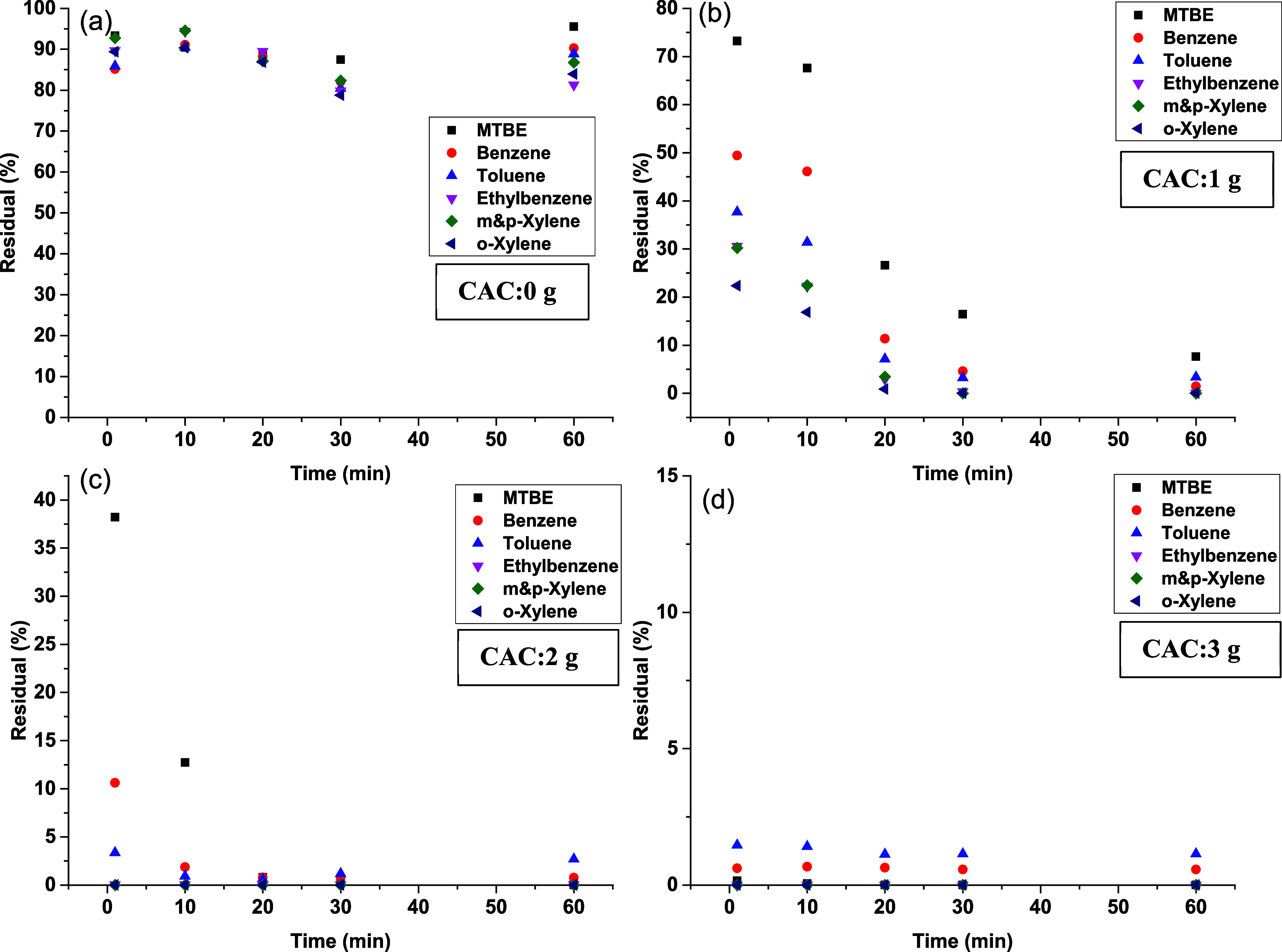
Residual concentrations of MTBE and BTEX at various CAC dosages: (a) control (0 g of CAC), (b) 1 g of CAC, (c) 2 g of CAC, and (d) 3 g of CAC.

The residual concentrations of MTBE and BTEX at different dosages are listed in Figure 4. The residual concentrations in the control sample (without CAC), as depicted in Figure 4a, indicate minor losses of MTBE and BTEX due to adsorption by soil or volatilization during the experiment. Similar results were reported by Alshahrani et al.,^44^ where soil had an insignificant effect on the adsorption of CAC.

After adding 1 g of CAC (Figure 4b), the residual concentrations of MTBE, benzene, toluene, ethylbenzene, and xylene dropped from 73, 50, 40, 30, and 50% at 1 min to 7, 1.4, 3.4, 0, 0, and 0 at 60 min, respectively. This demonstrates the higher affinity of the CAC for BTEX compared to MTBE. This can be attributed to strong competition between MTBE and BTEX on the limited available active sites on the adsorbent. The lower affinity of the CAC for MTBE compared to BTEX can be explained by the difference in their molecular characteristics. The MTBE molecule is small and more polar, leading to higher solubility in water. In contrast, BTEX compounds have higher hydrophobicity and stronger π-interactions with the carbon surface due to their aromatic structure, which enhances their removal efficiency.

At a higher dosage of 2 g (Figure 4c), the residual concentrations of BTEX and MTBE were below the detection limit for all time intervals, except for benzene, toluene, and MTBE, where more than 90% were removed in the first 10 min, while less than 1% was observed thereafter. This indicates the rapid adsorption of MTBE and BTEX onto the CAC. Increasing the CAC to 3 g, as shown in Figure 4d, led to increased removal efficiency for both MTBE and BTEX with near-complete removal. This confirms the availability of more active sites on the CAC at higher dosages, reducing the competition between MTBE and BTEX and thereby improving their removal efficiency. The maximum adsorption capacities were 385.49 μg/g for MTBE, 215 μg/g for benzene, 170 μg/g for toluene, 27.1 μg/g for ethylbenzene, and 108.97 μg/g for xylene. These findings are consistent with the results reported by Alshahrani et al.,^44^ where the CAC exhibited a higher affinity for BTEX compared to MTBE. However, their study reported a maximum removal of only 20% for MTBE and 40% for benzene and toluene. In contrast, the present study achieved near-complete removal for both MTBE and BTEX. These findings demonstrate the efficiency of the CAC for in situ remediation of water contaminated with MTBE and BTEX.

### 3.3 Core Flooding Adsorption Study

#### 3.3.1 Consolidated Carbonate System without CAC (Control)

**Figure 5:**
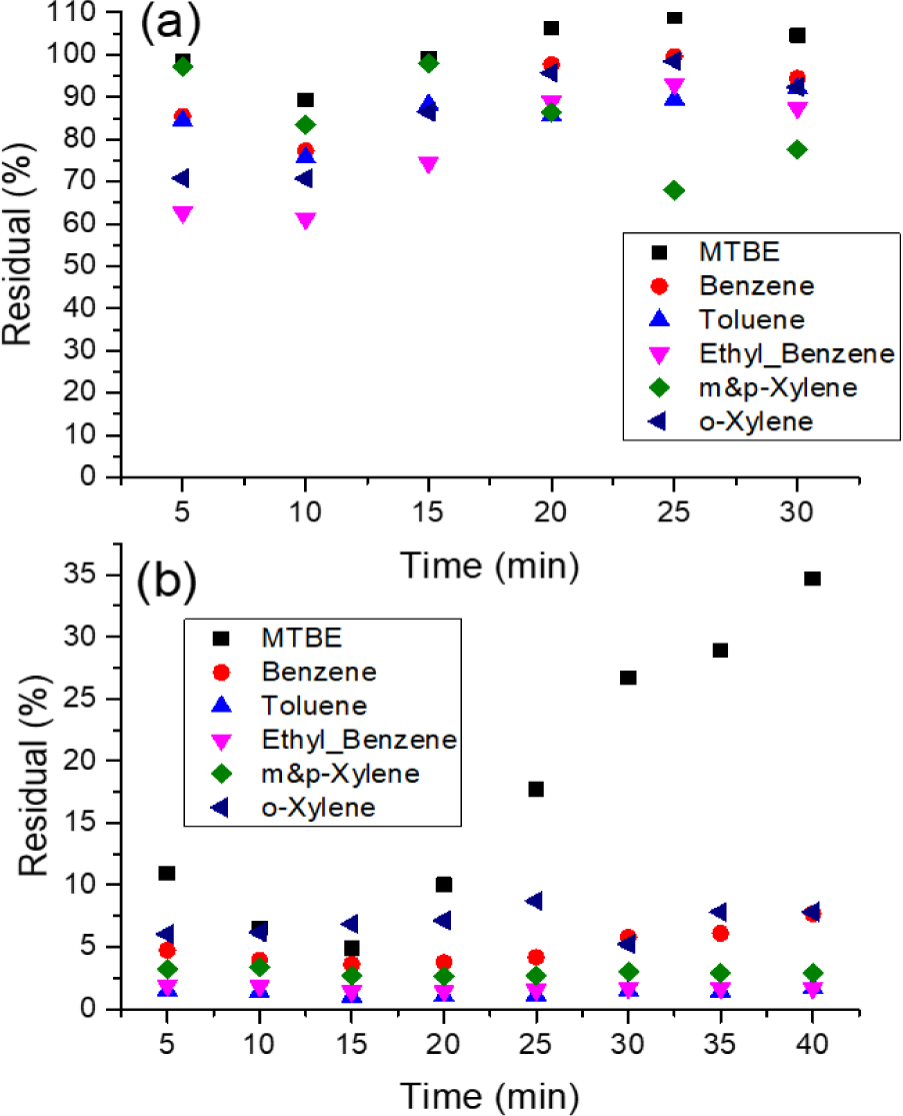
Residuals of MTBE, benzene, toluene, and xylene in the produced effluents from consolidated carbonate at a flow rate of 1 mL/min: (a) without using any CAC and (b) treated with CAC.

A control experiment was performed by injecting the spiked water sample into an Indiana consolidated carbonate sample, without using any CAC. The control experiment aims to assess the losses of contaminants due to the system before injection with CAC. Also, it will help in capturing the reductions in contaminant content that have been adsorbed by CAC. The control experiment showed reasonable hydrocarbon losses, where the concentrations of the produced effluent were very close to the hydrocarbon content of the injected water. The initial concentrations in the spiked water sample were 2000 ppb of MTBE, 1000 ppb of each benzene and toluene, 200 ppb of each ethylbenzene and *o*-xylene, and 300 ppb for *p*- and *m*-xylene. The injection of spiked water samples was conducted at a rate of 1.0 mL/min. Treated water samples were collected after intervals of 5, 10, 15, 20, 25, and 30 min and analyzed for the content of MTBE/BTEX. Figure 5a shows the concentrations of the aforementioned contaminants after passing through the system packed with consolidated carbonate materials without CAC. The obtained results indicate minor losses for MTBE, benzene, ethylbenzene, and *o*-xylene primarily due to their volatility and/or adsorption by the materials.

#### 3.3.2 Consolidated Carbonate Formations Treated with CAC

In this experiment, CAC was injected into an Indiana consolidated carbonate material. Then, a spiked water sample was injected into the consolidated carbonate material soaked with CAC. The results of the experiment are presented in Figure 5b, which shows the contaminants’ concentrations after introducing CAC into the system. Around 10 mL of the CAC was injected into the consolidated carbonate sample. Similar experimental conditions were applied in terms of injection rate (1 mL/min), which allowed the same contact time between the injected water and CAC.

A minimum MTBE content of 98 ppb was obtained after 15 min of injection; however, the MTBE concentration increased significantly until it reached around 700 ppb after 40 min. This shows that the amount of CAC used in the experiment is saturated when adsorbing MTBE. However, CAC was not saturated when adsorbing BTEX compounds. A removal efficiency of 95% was achieved by using a 1:1 ratio between the CAC and water spiked with MTBE and BTEX. This removal efficiency reduces to 65% once the ratio of CAC to water is 1 to 4. On the other hand, the removal of benzene and other hydrocarbons showed very good performance, where around 93% of the hydrocarbons were removed. Overall, using an injection rate of 1 mL/min resulted in the removal of 93 to 95% of the injected hydrocarbons by using a 1:1 ratio for the CAC and polluted water. The removal efficiency can be further improved by reducing the injection rate to allow for more contact time between the hydrocarbons and CAC.

#### 3.3.3 Impact of the Injection Flow Rate on the Removal Efficiency

**Figure 6:**
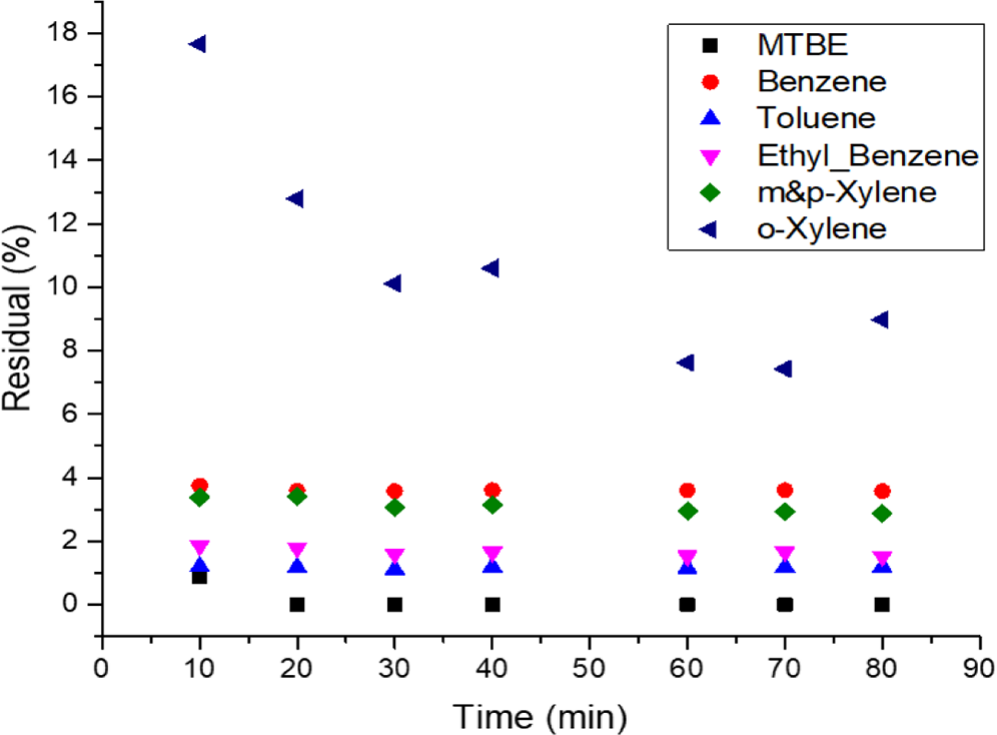
Residuals of MTBE and BTEX in the treated samples using consolidated carbonate treated with CAC at a flow rate of 0.5 mL/min.

In this experiment, the impact of the injection rate on the removal of MTBE, benzene, toluene, and xylene was investigated. The water solution was injected at a flow rate of 0.5 mL/min, and the residual hydrocarbon contents were measured, as shown in Figure 6. Reducing the injection rate showed considerable improvement in the removal efficiency for all contaminants. The residual MTBE concentration is almost zero, indicating 100% removal efficiency. The residual benzene concentration is 36 ppb, indicating that more than 96% of benzene was removed. Overall, using a lower flow rate led to efficient removal of all hydrocarbons due to the larger contact time between the injected water and CAC.

### 3.4 MTBE and BTEX Removal in Sandstone Formations

#### 3.4.1 Sandstone System without CAC

**Figure 7:**
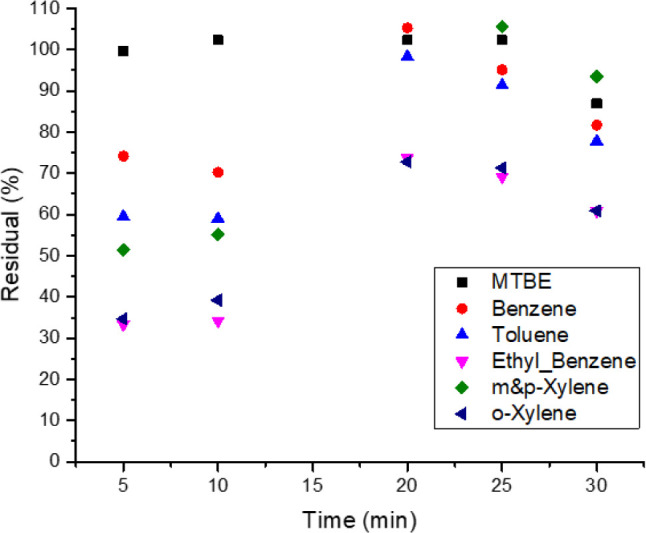
Residuals of MTBE, benzene, toluene, and xylene in the produced effluents from consolidated sandstone, without CAC. The flow rate is 1 mL/min.

The impact of bed material mineralogy on the removal efficiency was studied. A series of flooding experiments were conducted using consolidated sandstone. Figure 7 shows the residual hydrocarbon content from the control experiment to indicate losses within the system. Similar to the findings with carbonate materials, the results with sandstone materials revealed minor losses for MTBE and BTEX compounds, where the residual hydrocarbons were very close to the injected amount.

#### 3.4.2 Sandstone Formation Treated with CAC

**Figure 8:**
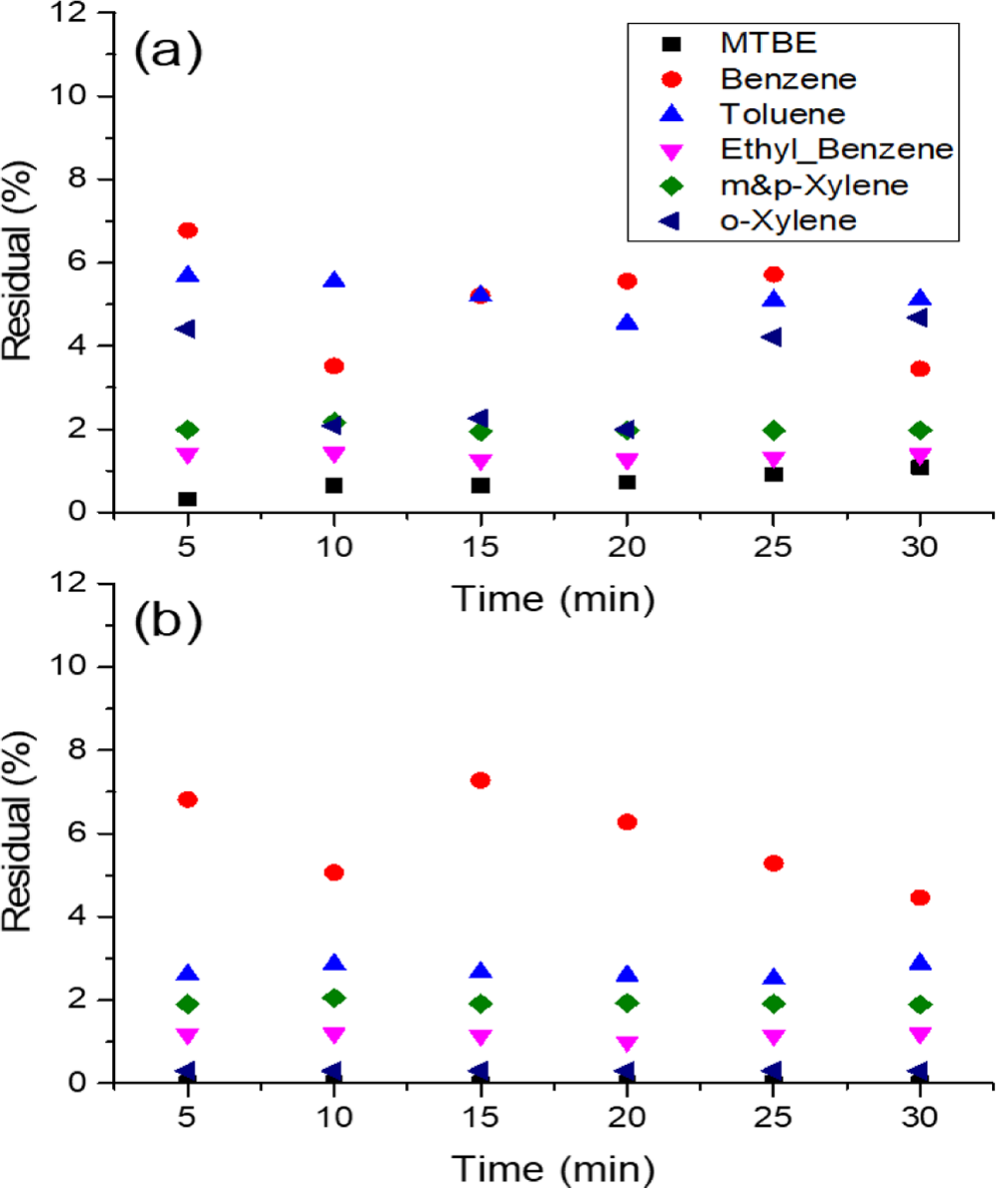
Residuals of MTBE and BTEX in the treated samples using consolidated sandstone treated with CAC at a flow rate of (a) 1 and (b) 0.5 mL/min.

In this experiment, the effect of using CAC as an adsorbent was examined by injecting the spiked samples at flow rates of 1.0 and 0.5 mL/min, with the results shown in Figure 8a,b, respectively. In Figure 8a, the average residuals of MTBE and benzene are 1 and 5%, respectively, indicating a removal efficiency of more than 95%. Subsequently, the impact of providing more contact time was investigated by running the flooding experiment at a flow rate of 0.5 mL/min (Figure 8b), resulting in a removal efficiency of 100% for MTBE, with a residual MTBE content near zero, while a removal efficiency of 93.8% was achieved for benzene.

A comprehensive comparative analysis was conducted to integrate all of the flooding results. Tables 3 and 4 present the residual concentrations of the MTBE/BTEX compounds in treated water using carbonate and sandstone materials, respectively. Two injection flow rates (0.5 and 1.0 mL/min) were employed to study the effect of treatment duration on performance. Reducing the flow rate from 1 to 0.5 mL/min resulted in improved removal efficiency, as it allowed more time for the CAC to adsorb a greater amount of the hydrocarbons. Additionally, the use of sandstone materials resulted in lower residual levels, indicating that the CAC is more efficient in sandstone formation. The higher performance of sandstone consolidated materials compared to carbonate materials may be attributed to several factors. Wettability could be a major factor influencing the treatment performance. The adsorption of CAC onto the carbonate surface may reduce the available surface area for hydrocarbons. Additionally, pore geometry and shape play a significant role; sandstone particles typically exhibit uniform and regular shapes, allowing better distribution of flowing water along the CAC. On the other hand, carbonate materials are characterized by complex pore systems. Consequently, the contaminated water may encounter restricted flow paths along the carbonate, leading to a reduction in the contact area between CAC and hydrocarbons.

**Table 3:** A Summary of the Residual Hydrocarbon Content in the Treated Water Using Consolidated Carbonate and Two Different Flow Rates

Component Name	Flow rate = 1mL/min			Flow rate = 0.5mL/min		
	CAC:Treated water					
	1:1	1:2	1:3	1:1	1:2	1:3
MTBE (ppb)	130.3	200.7	534.0	0.1	0.0	0.0
benzene (ppb)	39.4	37.5	57.7	35.9	36.1	36.0
toluene (ppb)	14.1	10.6	14.8	11.8	11.9	11.5
ethylbenzene (ppb)	3.7	2.9	3.4	3.6	3.3	3.1
*m*- and *p*-xylene (ppb)	10.2	8.0	9.0	10.2	9.4	8.9
*o*-xylene (ppb)	18.6	21.4	15.7	38.4	31.8	22.8

**Table 4:** A Summary of the Residual Hydrocarbon Content in the Treated Water Using Consolidated Sandstone and Two Different Flow Rates

Component Name	Flow rate = 1mL/min			Flow rate = 0.5mL/min		
	CAC:Treated water					
	1:1	1:2	1:3	1:1	1:2	1:3
MTBE (ppb)	12.8	14.4	21.6	ND	ND	ND
benzene (ppb)	35.2	55.6	34.5	50.6	62.8	44.6
toluene (ppb)	55.5	45.4	51.2	28.6	25.9	28.7
ethylbenzene (ppb)	2.9	2.5	2.8	2.4	2.0	2.4
*m* and *p*-xylene (ppb)	6.5	5.9	5.9	6.2	5.8	5.7
*o*-xylene (ppb)	6.2	6.0	14.1	ND	ND	ND

Figures S1–S6 summarize the removal efficiencies of MTBE/BTEX by CAC using carbonate and sandstone consolidated materials at different volume ratios (CAC:treated solution). The results indicate that removal efficiencies in carbonate and sandstone at 0.5 mL/min exceeded 95%.

## 4 Conclusions

The potential removal of MTBE and BTEX from water using CAC in batch and continuous modes was studied. For continuous flow, the core flooding system was investigated under different geological formations at two flow rates to simulate the subsurface conditions. The findings of this investigation revealed promising adsorption of CAC for MTBE and BTEX from water. The batch experiments revealed that the complete removal of MTBE and 99% removal of BTEX were achieved. Maximum adsorption capacities were 385.49 μg/g for MTBE, 215 μg/g for benzene, 170 μg/g for toluene, 27.1 μg/g for ethylbenzene, and 108.97 μg/g for xylene. The continuous flow using core flooding revealed insights into the factors that impact the CAC performance for contaminant adsorption in the subsurface. The obtained results demonstrated that the flow rate and geoformation significantly influenced MTBE and BTEX adsorption. The CAC exhibited higher removal efficiencies for MTBE and BTEX in sandstone compared to carbonate, attributed to the uniform and regular shapes of sandstone particles, allowing better distribution of the CAC. Moreover, a lower flow rate (0.5 mL/min) showed higher removal efficiencies compared to 1 mL/min, indicating the significance of contact time between the CAC and contaminated water. Complete removal of MTBE and over 95% removal of benzene and toluene was achieved under the best conditions (sandstone geoformation, 0.5 mL/min flow rate). The CAC demonstrated excellent MTBE and BTEX removal capabilities, with efficiencies exceeding 95%, offering a promising adsorbent for in situ groundwater remediation, particularly in sandy aquifers. Further investigations are needed to optimize the amount of CAC for large-scale in situ applications.

## Supporting Information Available

The removal efficiencies of MTBE/BTEX by CAC using carbonate and sandstone consolidated materials at different volume ratios (CAC:treated solution) under different flow rates (PDF)

## Abbreviation

BET: Brunauer, Emmett, and Teller
BTEX: benzene, toluene, ethylbenzene, and xylenes
CAC: colloidal activated carbon
DI: deionized water
EDXS: energy dispersive X-ray spectroscopy
EPA: Environmental Protection Agency
FTIR: Fourier transform infrared spectroscopy
GC/MS: gas chromatography/mass spectrometry
IARC: International Agency for Research on Cancer
MCLs: maximum contaminant levels
MTBE: methyl tertiary-butyl ether
PFAS: per- and poly-fluoroalkyl substances
SEM: scanning electron microscopy
USEPA: United States Environmental Protection Agency
VOCs: volatile organic compounds
XRD: X-ray diffraction
