# Supplementary material for: Removal of MTBE and BTEX Pollutants from Contaminated Water Using Colloidal Activated Carbon (CAC)
Source: ACS Omega. 2024 Dec 26;10(1):509–19. doi: 10.1021/acsomega.4c06974 (PMC11739941; doi:10.1021/acsomega.4c06974)
Supplement: Supplementary file 1 — ao4c06974_si_001.pdf [file ao4c06974_si_001.pdf]

Removal of MTBE and BTEX Pollutants from Contaminated Water Using Colloidal Activated Carbon (CAC)

Abdullah Basaleh[1,2], Amjed Hassan[2,3], Bassam Tawabini[1,2*], Mohamed Mahmoud[2,3], Fahad Alghamdi[4], Abdulmogni Althubiti [4]. Muhammad Alrayaan[4] and Rayan Al-Nasser[4]

[1] Geosciences Department, College of Petroleum Engineering and Geosciences P.O. Box 5070, King Fahd University of Petroleum & Minerals (KFUPM), Dhahran 31261, Saudi Arabia.

[2] Center for Integrative Petroleum Research, College of Petroleum Engineering and Geosciences P.O. Box 5070, King Fahd University of Petroleum & Minerals (KFUPM), Dhahran 31261, Saudi Arabia.

[3] Petroleum Engineering Department, College of Petroleum Engineering and Geosciences P.O. Box 5070, King Fahd University of Petroleum & Minerals (KFUPM), Dhahran 31261, Saudi Arabia.

[4] Groundwater Protection Unit, Environmental Department, Saudi Aramco P.O. Box1977, Dhahran 31311, Saudi Arabia.

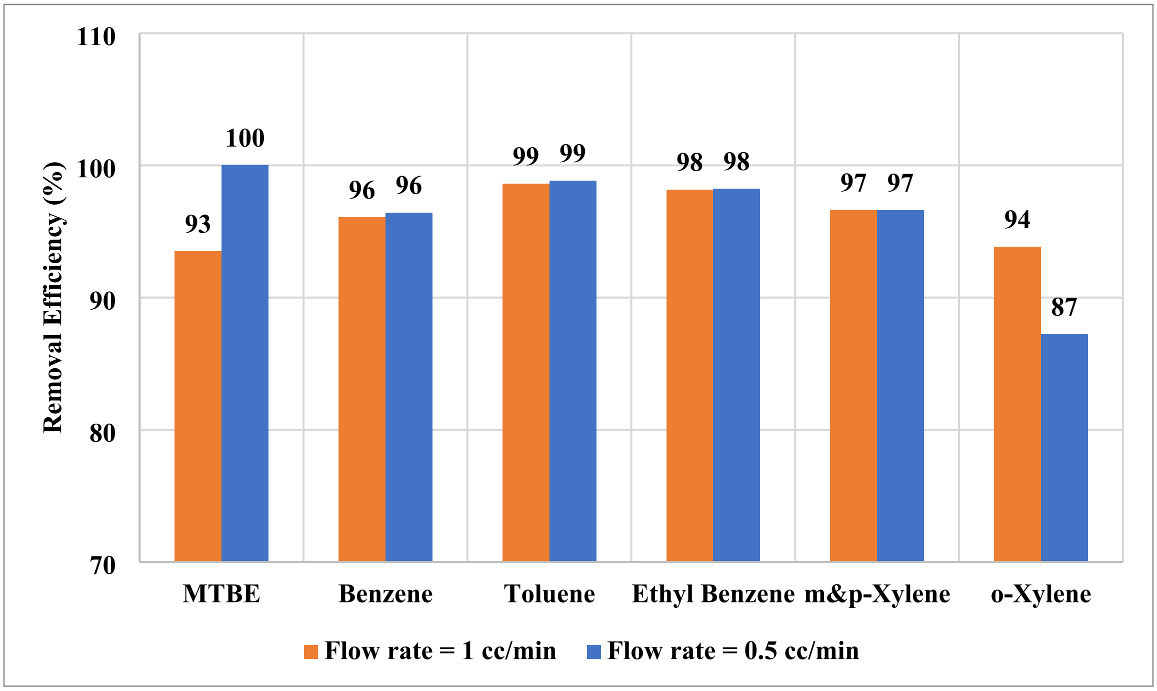

Figure S 1:The removal efficiency using a 1 to 1 ratio between CAC and polluted water, utilizing consolidated carbonate material.

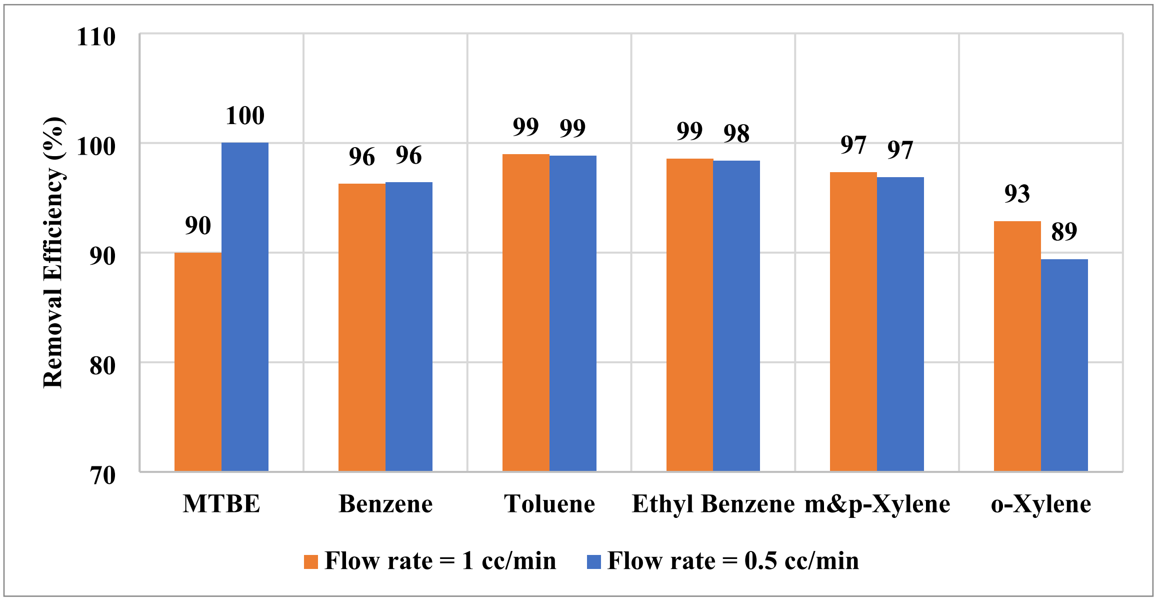

Figure S 2:The removal efficiency using a 1 to 2 ratio between CAC and polluted water, utilizing consolidated carbonate material.

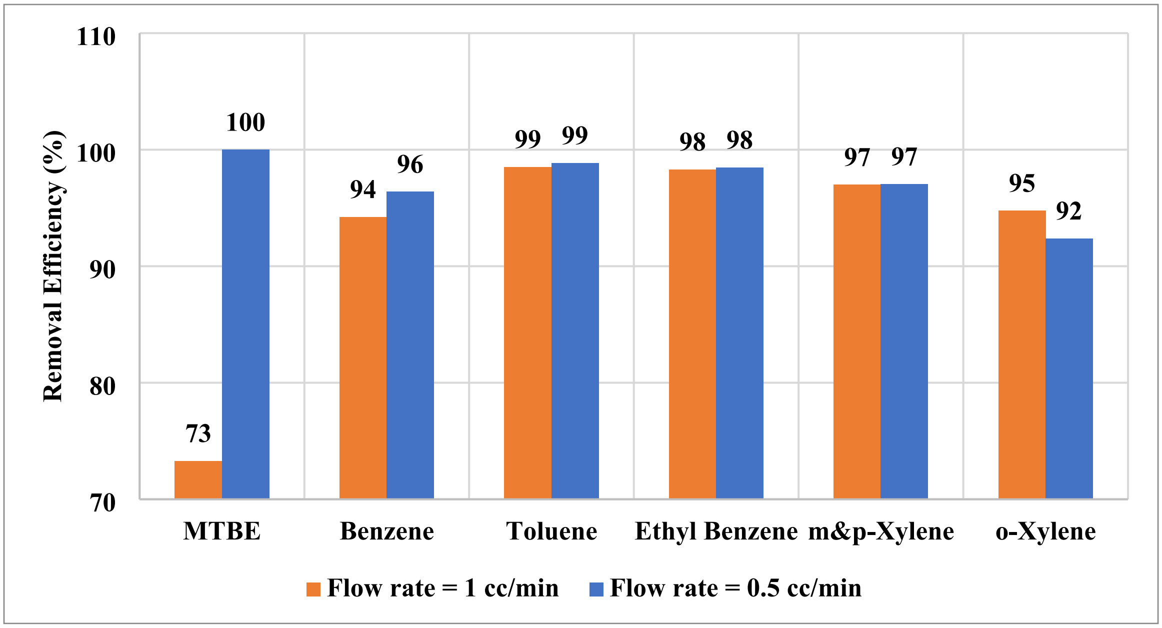

Figure S 3:The removal efficiency using a 1 to 3 ratio between CAC and polluted water, utilizing consolidated carbonate material.

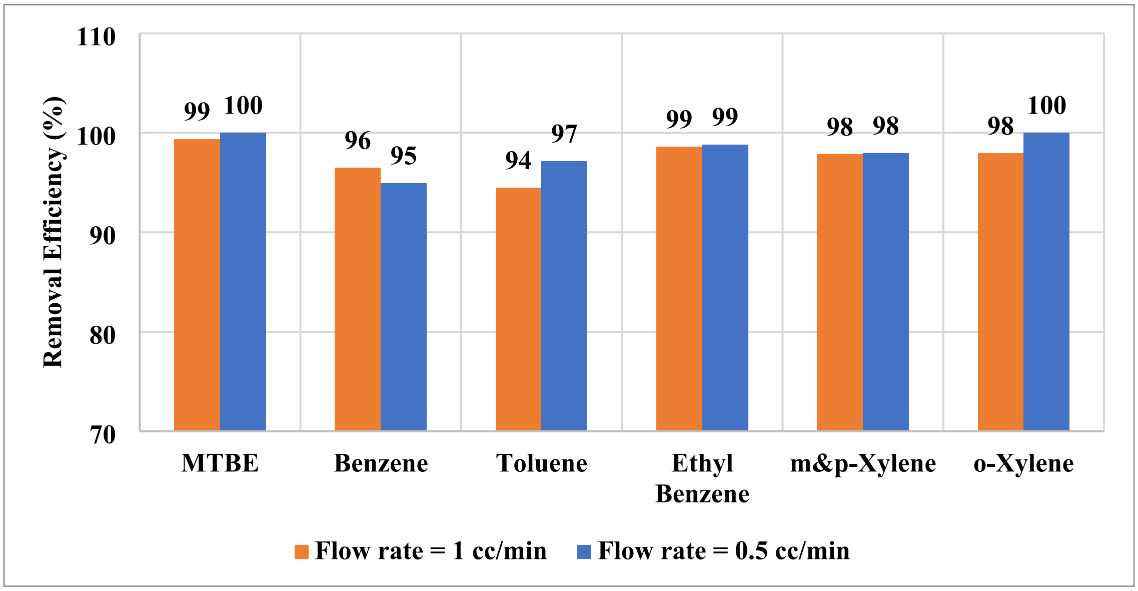

Figure S 4:The removal efficiency using a 1 to 1 ratio between CAC and polluted water, utilizing consolidated sandstone material.

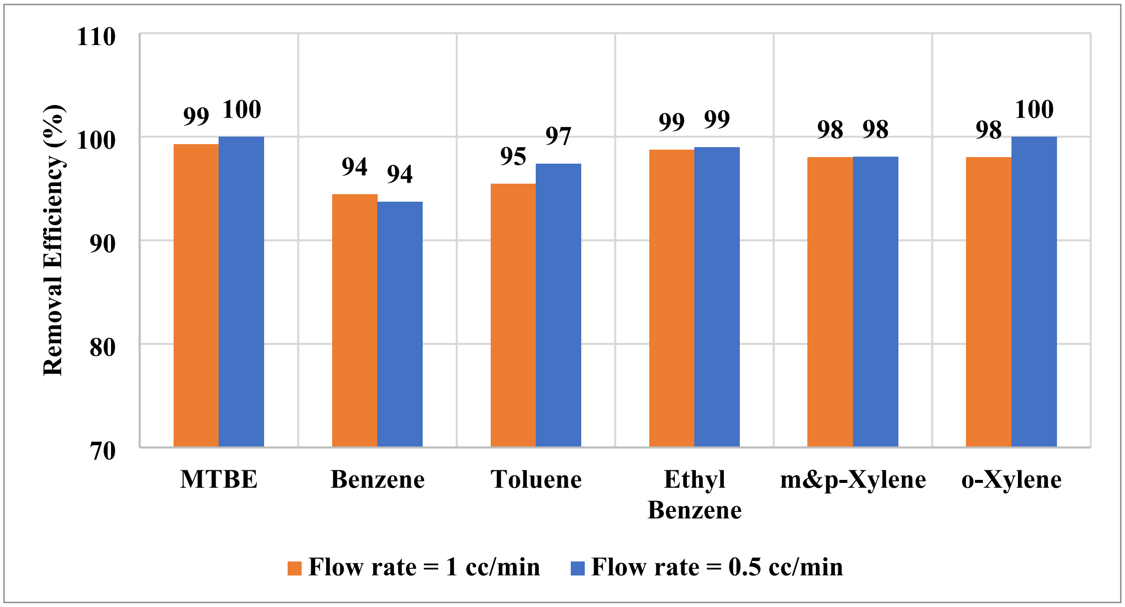

Figure S 5:The removal efficiency using a 1 to 2 ratio between CAC and polluted water, utilizing consolidated sandstone material.

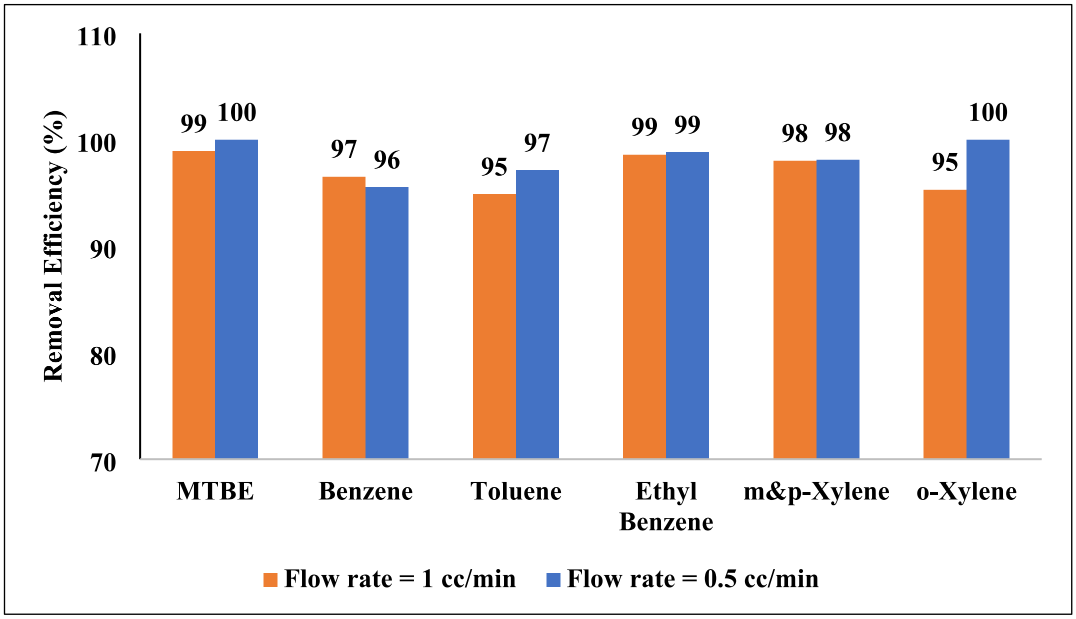

Figure S 6:The removal efficiency using a 1 to 3 ratio between CAC and polluted water, utilizing consolidated sandstone material.
